# A Clinical Approach to Detecting Germline Pathogenic Variants From Tumor-Only Sequencing

**DOI:** 10.1093/jncics/pkaa019

**Published:** 2020-03-05

**Authors:** Ozge Ceyhan-Birsoy, PhD, Maksym Misyura, Diana Mandelker

**Affiliations:** Department of Pathology, Memorial Sloan Kettering Cancer Center, New York, NY, USA

In this issue of *JNCI Cancer Spectrum*, Klek and colleagues (1) describe a single institution’s approach to identifying patients who carry a genetic cancer predisposition from tumor-only sequencing. They demonstrate that careful review of tumor-sequencing data substantially increased the percentage of cancer patients in their cohort diagnosed with a hereditary cancer susceptibility.

Tumor-only profiling by next-generation sequencing has been widely implemented in oncology practices to help guide therapeutic decisions, clarify diagnosis, and aid in prognostication. This approach has been adopted by both academic centers and commercial companies alike (2–5). Although tumor-only sequencing provides a cost-effective approach to identifying somatic variants present in the tumor, it will also detect any germline variants present in all cells of the body. Importantly, it can be difficult to distinguish somatic and germline alterations from tumor-sequencing data without a normal matched control (eg, blood sample) (6–8).

Klek et al. demonstrate that involvement of clinical genetics in a tumor-only sequencing data review process can improve the identification of patients and families with germline pathogenic variants in hereditary cancer predisposition genes. After implementing a formal tumor-only sequencing data review and genetic counseling referral process, the percentage of patients undergoing tumor-only sequencing with a detected pathogenic germline variant increased from 1.4% to 7.5%. Their findings are in agreement with recent studies demonstrating that the inclusion of germline genetics expertise in tumor-sequencing analysis improves the identification of germline cancer risk in various cancer-patient populations (6,9). Notably, some of these individuals would not have met standard criteria for germline testing, and the only indication for genetic testing was a variant identified on tumor-only sequencing.

Estimating the possibility of a variant having germline vs somatic origin can be complex and requires an expert review of multiple factors, including variant attributes in sequencing data and the prior probability for the individual to have a germline pathogenic variant in a given gene. A few automated approaches to tumor-sequence analysis have been developed to predict which variants are likely to be germline in origin, and guidelines have been proposed for implementing such variant filters in laboratory practices (10). However, although these methods can detect a substantial proportion of germline pathogenic variants from tumor-sequencing data, any automated method will have some limitations because of the nature of the tumor genome. For example, variants with allele fractions close to 50% are assumed likely to be germline heterozygous variants, but this can be complicated by tumor purity and changes in tumor allele fractions because of somatic deletion and amplification events. Moreover, from a clinical perspective, assessing the prior probability of a patient having a hereditary cancer predisposition syndrome can also be difficult. Although genes such as *NF1* or *TSC1* are believed to have high penetrance, many patients may have milder presentations and may not be diagnosed until examined specifically for associated features. Several studies have shown that this is even more complex for diseases caused by lower penetrance genes, such as hereditary breast and ovarian cancer and Lynch syndrome. Guideline-directed genetic testing misses a substantial proportion of patients with pathogenic germline variants for these disorders (11–14). Having genetic counseling involvement in the review of tumor-sequencing results helps in multiple steps of the process to accurately identify and classify germline pathogenic variants, as well as clarify their implications.

Klek et al. show that the review of tumor-sequencing data by a molecular tumor board increases the yield for detecting pathogenic germline variants and that this methodology can contribute to detecting hereditary cancer susceptibilities in individuals who otherwise may not have had genetic testing. However, as the authors suggest, most tumor-only sequencing panels do not provide complete coverage of all target genes and are limited in their ability to detect certain variant types such as exon-level copy number variants and variants in high homology regions. Therefore, it should be recognized that tumor sequencing is not a substitute for clinical genetic testing where the gene panels are designed and validated specifically for germline variant detection, and all variants are scrutinized and interpreted according to American College of Medical Genetics and Genomics criteria (15). Moreover, although the criteria developed by Klek et al. to help identify variants of potential germline origin are well-thought-out heuristic methods, the only unambiguous method to immediately distinguish between germline and somatic origin is matched tumor-normal sequencing (12,16,17). However, the cost and challenges of coordinating a paired analysis may be limitations to its implementation in many institutions.

In this study, Klek et al. (1) clearly demonstrate that tumor sequencing can provide an opportunity to detect germline pathogenic variants if a proper system of manual review or automated flagging of variants on tumor-sequencing reports is implemented. Additionally, any institution that implements a workflow for identifying variants from tumor-sequencing reports with a high index of suspicion for germline origin must also have genetics professionals available to interpret these results for patients and provide expert genetic counseling for patients and their families. Such a comprehensive review process of tumor-sequencing data can help identify cancer patients who harbor a previously undiagnosed hereditary cancer predisposition.

## Notes

The authors declare no conflicts of interest.
